# Adaptive and maladaptive features of schizotypy clusters in a community sample

**DOI:** 10.1038/s41598-021-95945-0

**Published:** 2021-08-17

**Authors:** Bertalan Polner, Ernő Hupuczi, Szabolcs Kéri, János Kállai

**Affiliations:** 1 Department of Cognitive Science, Budapest University of Technology and Economics, Budapest, Hungary; 2 Cognitive Neuroscience Research Group, Medical Faculty, Institute of Behavioral Sciences, University of Pécs, Pecs, Hungary; 3 National Institute of Psychiatry and Addictions, Budapest, Hungary; 4 Department of Physiology, University of Szeged, Szeged, Hungary

**Keywords:** Psychology, Human behaviour, Stress and resilience

## Abstract

Schizotypal personality traits correlate with psychopathology and impaired functional outcome. Yet advantageous aspects of positive schizotypy may exist which could promote resilience and creativity, and several studies have identified a high positive but low negative schizotypy group with some signs of adaptation. The aim of our study was to clarify whether such individuals demonstrate only traits associated with well-being, or they also have traits that predict impairment. Participants (N = 643 students, 71.5% female) completed measures of schizotypy, resilience, self-esteem, self-concept clarity, and absorption. We identified four clusters: an overall low schizotypy, an overall high schizotypy, a disorganised-interpersonal schizotypy and a positive schizotypy cluster. The overall high schizotypy cluster seemed to be the most vulnerable as it was the least resilient and showed widespread maladaptation, whereas the high positive schizotypy cluster had intact self-esteem and high resilience and its elevated absorption may hold the promise for adaptive outcomes such as creativity and positive spirituality. However, the high positive schizotypy cluster lacked self-concept clarity. The results suggest that individuals showing high positive and low negative schizotypy demonstrate features promoting mental well-being to an extent that is higher than in all the other clusters, while their self-concept impairment is similar to that observed in the high and the disorganised-interpersonal schizotypy clusters. Better understanding of these factors could be informative for prevention and treatment of psychosis-spectrum disorders.

## Introduction

Schizotypy is a set of personality traits in the general population that can be seen as attenuated symptoms of schizophrenia and show overlap with schizophrenia at multiple levels of analysis^1–4^. Schizotypy has not only been conceptualised as a personality trait emerging from interactions between genetic risk for schizophrenia and the environment^5–7^, but also as an aspect of variation between healthy individuals that may also be advantageous in certain contexts^8–10^. Development of psychotic disorders in people with high schizotypy is theorised to be a function of intrapersonal psychological dynamics, various traits (such as intelligence, anhedonia, introversion or anxiety proneness), and favourable vs. adverse social circumstances, and such ideas have received some empirical support^5–8,11–16^.

Positive, negative, and disorganised dimensions of schizotypy can be distinguished (but also see^17–24^) and they show specific associations with psychopathology and functional outcome. For instance, positive schizotypy predicts (social) anxiety^25–27^ and depression^26–28^, psychotic-like, paranoid and schizotypal symptoms^27,29^, substance abuse and history of mental health treatment^28^, and more negative self and other schemas^27^. In turn, negative schizotypy is associated with negative and schizoid symptoms, less positive self and other schemas^27^, and lack of intimate relationships^28^. Moreover, both positive and negative schizotypy are associated with poor social adjustment^28^, and reduced quality of life, with negative schizotypy showing a stronger effect^30–32^. Last but not least, disorganised schizotypy correlates with poor mental health and insomnia^33^, and predicts impaired cognitive control, increased emotionality and emotional confusion^34^. In daily life, disorganisation is associated with heightened negative and impoverished positive affect^35^.

Complementing the above-mentioned correlational approach to schizotypy, another line of research has examined latent schizotypy groups. Typically, these studies reported clusters that scored either low or high on positive, negative, and disorganised dimensions of schizotypy, which we will label as *low schizotypy* and *high schizotypy* groups, respectively. Furthermore, a group characterised by high positive but low negative schizotypy was also often detected—we will refer to them as the *positive schizotypy* group^36–40^. This latter group shows various signs of psychotic and affective psychopathology^36,37^, however, it also possesses potential sources of resilience such as elevated openness and extraversion^37^, low negative emotionality, and high cooperativeness and self-transcendence^39^ and increased sense of coherence^41^. Moreover, enhanced motivational functioning and hedonic capacity in the positive schizotypy group is implicated by findings showing that it has very low levels of negative schizotypy^38^, has an increased capability of experiencing anticipatory and consummatory pleasure, and it is characterised by lower suppression and higher expression of emotions^36^. In contrast, the overall high schizotypy cluster has the worst functional outcome^37^, shows high negative emotionality and reduced cooperativeness^39^, and demonstrates the poorest cognitive performance^38,39^.

Relatedly, several lines of evidence suggest that there might be adaptive aspects of positive schizotypy that may enrich one’s life with meaning and can be a source of resilience^42^. Positive schizotypy has been shown to covary with openness^43,44^, which is a robust predictor of creativity^45^, happiness, and quality of life^46^. On a related note, positive schizotypy has a weak positive correlation with creativity^47^, and more specifically, with the phenomenology of artistic creativity^48,49^. Moreover, once adverse correlates of disorganised schizotypy such as reduced intellect or insomnia are statistically adjusted for, disorganised schizotypy positively predicts creative achievements in science and problem solving on the remote associates task, respectively^33^. Another study has found that specific aspects of positive (magical thinking, odd beliefs) and disorganised schizotypy (odd behaviour) predicted higher subjective well-being, once the negative effects of interpersonal impairments were adjusted for^50^.

The motivation of the present study was to further elaborate the idea that positive schizotypy can be adaptive^42^ by a fine-grained analysis. First, we assessed traits relevant for creativity and psychopathology in context of the schizophrenia-spectrum such as absorption^44,51–53^ and self-concept clarity^54–58^, respectively. Absorption has been conceptualised as the tendency to get deeply immersed in sensory experiences and imagination while suspending a sense of active control^51^. It is associated with openness to experience, synesthetic experiences and appreciation and production of art^59^, and it predicts psychotic-like experiences^53^ and hallucinations^52^. Furthermore, absorption mediates the association of positive schizotypy with artistic creativity^49^ and predicts spiritual and aesthetic experiences^51^. Self-concept clarity is defined as ‘the extent to which the contents of …self-concept are clearly and confidently defined, internally consistent, and temporally stable’^54^ and it predicts mental well-being and psychological adaptation^54,55^. Furthermore, in line with theories emphasising the role of narrative self-disturbance in schizophrenia-spectrum disorders^60,61^, low self-concept clarity predicts thought disorder^62^ and schizotypy and psychotic-like experiences^56–58^. Second, to gain information on mental health and well-being more broadly, we measured resilience^63^ and self-esteem^64^. Resilience is defined as the ability to successfully cope with stressors and adapt when faced with adversity^65^. It has been argued that the interaction between resilience and schizotypy is critical in predicting risk for schizophrenia in that individuals with high schizotypy and low resilience are the most vulnerable^4^. On the other hand, identifying individuals with high schizotypy and high resilience can facilitate the understanding of protective factors. Self-esteem is a general indicator of well-being and it correlates with school and occupational success, happiness and reduced depression^64^. Relatedly, previous studies have shown that positive and negative schizotypy are associated with more negative and less positive valuation of the self, respectively^27^.

It remains an open question whether some manifestations of positive schizotypy are linked to increased well-being instead of impaired mental health. We argue that two versions of an adaptive schizotypy hypothesis can be formulated. Both predict a positive schizotypy group with certain adaptive features that can promote well-being and creativity. However, the strong version claims that positive schizotypy in itself can be benign and thus it would predict no psychological maladaptation in this group^39,42^. In contrast, given the specific associations between positive schizotypy and various mental health complaints^26–29^, a moderate version also seems plausible: this would predict that the positive schizotypy group suffers from subtle psychopathology but also shows greater resilience and improved self-esteem, which effectively counter the effects of an incoherent self and ultimately contribute to increased quality of life. Here, we empirically compare these competing versions of the adaptive schizotypy hypothesis, using a large non-clinical sample and a unique set of highly relevant personality measurements. Considering developmental aspects of schizotypy^66^, and age and sex differences in the prevalence of schizotypal personality disorder and the pattern of comorbid disorders^67^, we also evaluate sex and age differences between the groups.

## Methods

### Participants

The sample comprised of 643 university students (71.5% female, mean of age = 25.7, SD = 7.9, min = 18, max = 49, skewness = 1.5, kurtosis = 1). Participants were invited to take part in a study on ‘Hungarian state of mind’. We have collected data with a convenience sampling method. The only inclusion criterion was to be aged between 18 and 49 years. Individuals with missing schizotypy or resilience data were excluded from the analysis. Participants were informed that the questionnaire battery included multiple questionnaires which have no correct or incorrect answers and were asked to briefly consider the questions and respond according to how they see themselves. Participation was voluntary and participants provided informed consent. The study was conducted in accordance with the Helsinki Declaration. The study is approved by the Institutional Review Board of the University of Pécs (ethical approval No. 6732 PTE/2017).

### Measurements

Descriptive statistics and internal consistency reliability estimates of the measurements are shown in Table 1. All reliabilities were good or excellent (0.82 < Cronbach’s α < 0.95).

**Table 1:** Descriptive statistics.

Variable	m	SD	mdn	Min	Max	sk	ku	N	α
Interpersonal schizotypy (SPQ-BR)	1.3	0.8	1.3	0.0	3.5	0.4	− 0.5	643	0.88
Cognitive-perceptual schizotypy (SPQ-BR)	1.0	0.6	0.9	0.0	3.1	0.7	0.5	643	0.82
Disorganised schizotypy (SPQ-BR)	1.5	0.8	1.4	0.0	4.0	0.6	− 0.2	643	0.88
Resilience (CD-RISC-25)	68.6	13.6	70.0	23.0	100.0	− 0.4	− 0.2	643	0.90
Absorption (TAS)	2.7	0.8	2.6	1.0	4.9	0.2	− 0.3	641	0.95
Self-concept clarity (SCCS)	3.9	0.9	4.2	1.1	5.0	− 0.9	0.1	641	0.92
Self-esteem (RSE)	3.0	0.6	3.0	1.0	4.0	− 0.3	− 0.5	639	0.90

*m* mean, *mdn* median, *sk* skewness, *ku* kurtosis.

Schizotypal personality traits were measured with the Schizotypal Personality Questionnaire-Brief Revised (SPQ-BR^68^; Hungarian adaptation^69^) that has 32 5-point Likert items. The SPQ-BR was developed by Cohen et al. to overcome the psychometric shortcomings of the Schizotypal Personality Questionnaire-Brief^70^, which has 22 true/false items, and its factor structure has been criticised. For the SPQ-BR, a structure with seven first-order and three second-order factors has repeatedly been confirmed^68,69^: this includes a Cognitive-Perceptual/positive schizotypy factor (magical thinking, unusual perceptions, suspiciousness/ideas of reference), an Interpersonal/negative schizotypy factor (constricted affect/no close friends and social anxiety), and a Disorganised factor (odd speech and eccentric behaviour). Here, we used subscale mean scores corresponding to the second-order factors (possible range 0–4). The construct validity of these scores is implicated by associations with self-reported family history of schizophrenia^71^ and psychosis symptoms^72^. Note that SPQ-BR scores indicate schizotypal traits that can be considered as a proxy to ‘true schizotypy’ (that is, a phenotype indicating genetic risk for schizophrenia).

Absorption was measured with the Tellegen Absorption Scale (TAS^73^; Hungarian adaptation^74^) which consists of 34 5-point Likert items. Self-concept clarity was examined with the Self-Concept Clarity Scale (SCCS^54^; Hungarian adaptation^75^) that includes 12 5-point Likert items. Again, we calculated a mean score (possible range 1–5). Resilience was assessed with the Connor-Davidson Resilience Scale (CD-RISC-25^65^; Hungarian adaptation^76^) contains 25 5-point Likert items that assess tenacity, control under stress, adaptation to and recovery after adversity and finding meaning in life events. We calculated a total score for this scale (possible range 0–100). The CD-RISC-25 total score is a valid indicator of resilience: it correlates with less perceived stress and disability, its change predicts therapeutic response^65^, and it also moderates the association between childhood emotional neglect and subsequent psychiatric symptoms^77^. Finally, participants also completed the Rosenberg Self-esteem (RSE^78^; Hungarian adaptation^79^) scale that contains 10 4-point Likert items that assess the subjective evaluative aspect of the self. We calculated an average score for this scale (possible range 1–4).

### Statistical analyses

Statistical analyses were performed with R (v3.5.2)^80^ in RStudio (v1.1.463)^81^. Data and the script for the analyses are available here: https://osf.io/m7hy2/. Prior to cluster analysis, we examined clustering tendency of the data with the Hopkins-statistic^82^, and also via visual inspection of the ordered dissimilarity image. If the data contains no structure, the Hopkins-statistic will be near 0.5, whereas values closer to zero indicate increasing clustering tendency.

First, we performed hierarchical cluster analysis using z-standardised cognitive-perceptual, interpersonal, and disorganised schizotypy subscale scores of the SPQ-BR. Based on previous studies, we planned to compare solutions involving 3 and 4 clusters. Dendrograms obtained with single, average, and complete linkage were visually inspected. We expected to find three or four clusters, characterised by: (a) overall low schizotypy, (b) overall high schizotypy, (c) high positive but low interpersonal and disorganised schizotypy, and (d) low positive but high interpersonal and disorganised schizotypy. We evaluated the goodness of clustering according to theoretical considerations and we also examined their internal statistical validity by computing connectivity, the Dunn-index, silhouette width^83^ and the S_Dbw index^84^. For descriptive purposes, we compared the clusters in terms of sex ratio and age.

Then, to determine the extent of adaptation vs. maladaptation in the positive schizotypy group, we compared the clusters in terms of resilience (CD-RISC-25), absorption (TAS), self-esteem (RSE) and self-concept clarity (SCCS). We performed Kruskal–Wallis tests, and if a test was significant, it was followed up by Mann–Whitney tests, with Cliff’s Delta calculated as effect size.

## Results

First, we examined whether there were groups in the dataset. Clustering tendency in the data was implicated by the Hopkins-statistics (average over 10 iterations was 0.32, min = 0.31, max = 0.33) and by visual inspection of the ordered dissimilarity image (Supplementary Fig. S1). After comparing the dendrograms yielded by different linkage methods, we chose complete linkage as it produced a relatively balanced solution, which contrasted with the strongly asymmetric single and average linkage dendrograms (Supplementary Fig. S2).

### Identifying the schizotypy clusters

We examined clustering solutions that yielded three and four clusters. Our aim was to find and characterise a positive schizotypy cluster, that is, a group of individuals showing high level of positive and low level of negative schizotypy.

**Figure 1:**
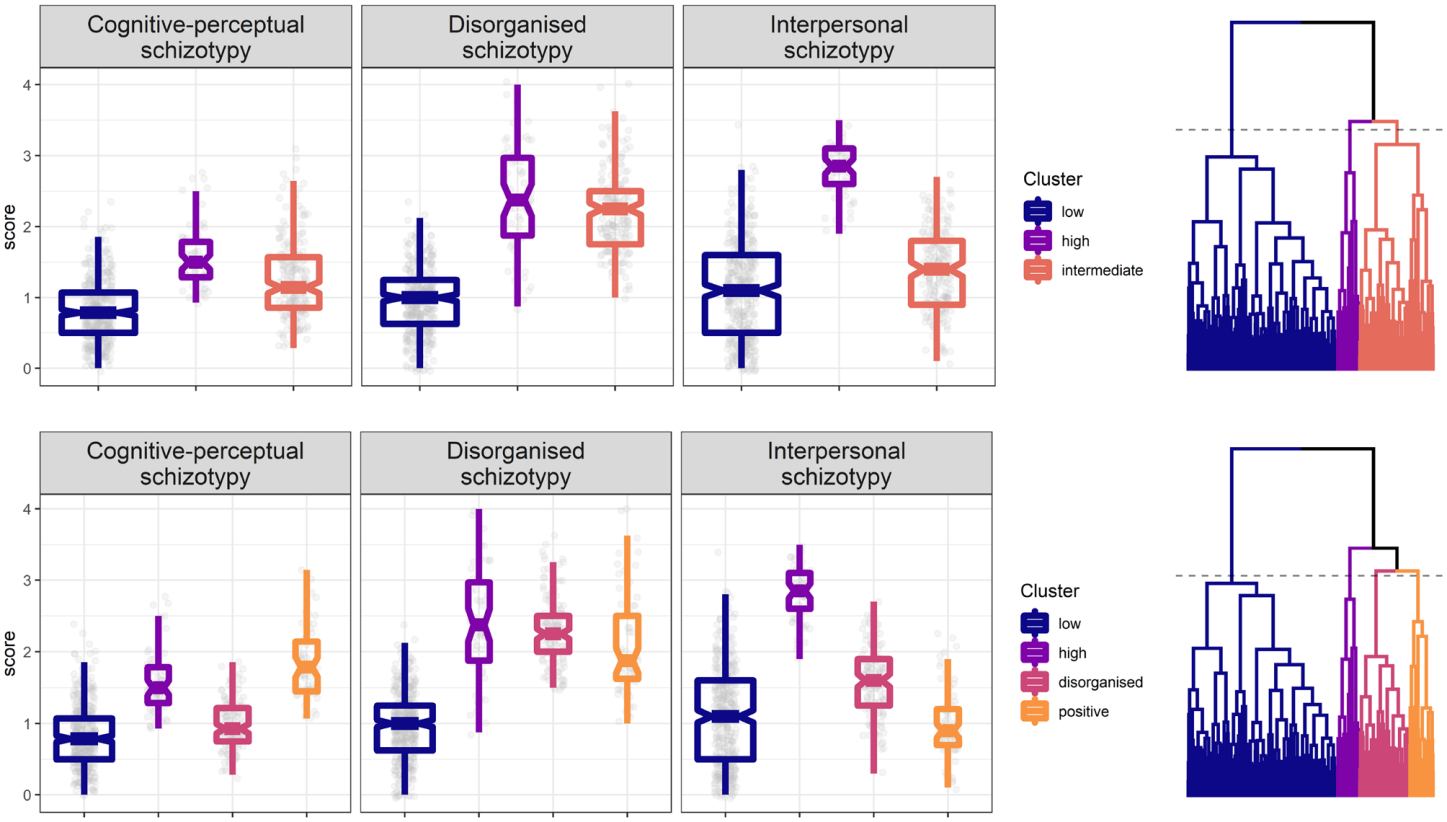
Results of hierarchical clustering after cutting the dendrogram at heights giving 3 (top row) and 4 (bottom row) clusters. Clustering was performed with complete linkage on the z-standardized scores of the Cognitive-perceptual, Disorganised, and Interpersonal subscales of the SPQ-BR. Note that the points are jittered to facilitate visibility.

The three-cluster solution yielded (1) a large cluster characterised by overall low schizotypy (N = 392, 61%), (2) a medium sized cluster characterised by intermediate levels of schizotypy (N = 193, 30%), and (3) a small cluster characterised by overall high schizotypy (N = 58, 9%) (Fig. 1, top row). The clusters differed significantly from each other in terms of all dimensions of schizotypy (Kruskal–Wallis *p* values < 0.001; Mann–Whitney *p* values < 0.001 and Cliff’s Delta values ranging from 0.29 to 0.96), except for the difference between cluster 2 and 3 on the Disorganised subscale of the SPQ-BR (*p* = 0.083; Cliff’s Delta = 0.15).

The four-cluster solution split the above mentioned medium sized intermediate schizotypy cluster into (3) one showing low positive, high disorganised and intermediate interpersonal schizotypy (N = 131, 20%), and (4) another showing high positive and intermediate disorganised schizotypy, but low interpersonal schizotypy (N = 62, 10%) (Fig. 1, bottom row). Again, the clusters significantly differed from each other in terms of all schizotypy dimensions (Kruskal–Wallis *p* values < 0.001; Mann–Whitney *p* values < 0.014; Cliff’s Delta values ranging from 0.26 to 0.99, see details in Table 2), with two exceptions: no significant difference was detected between cluster 1 and 4 on the Interpersonal subscale of the SPQ-BR (*p* = 0.66; Cliff’s Delta = 0.03), and between cluster 2 and 3 on the Disorganised subscale of the SPQ-BR (*p* = 0.284; Cliff’s Delta = 0.1).

**Table 2:** Descriptive statistics and comparison of the clusters.

Variable	Low	High	Disorg	Pos	χ^2^(3)	Low vs. high	Low vs. disorg	Low vs. pos	High vs. disorg	High vs. pos	Disorg vs. pos
*N* (%)	392 (61%)	58 (9%)	131 (20%)	62 (10%)							
Interpersonal schizotypy (SPQ-BR)	1.1 (1.1)	2.8 (0.5)	1.6 (0.6)	0.9 (0.5)	203.4	− 0.96*** [− 0.99; − 0.9]	− 0.45*** [− 0.53; − 0.36]	0.03 [− 0.1; 0.16]	0.95*** [0.9; 0.97]	0.99*** [0.97; 1]	0.59*** [0.43; 0.72]
Cognitive-perceptual schizotypy (SPQ-BR)	0.8 (0.6)	1.5 (0.5)	0.9 (0.5)	1.8 (0.7)	217.9	− 0.78*** [− 0.85; − 0.68]	− 0.27*** [− 0.37; − 0.17]	− 0.89*** [− 0.95; − 0.74]	0.72*** [0.6; 0.81]	− 0.36*** [− 0.53; − 0.15]	− 0.87*** [− 0.92; − 0.79]
Disorganised schizotypy (SPQ-BR)	1 (0.6)	2.4 (1.1)	2.2 (0.5)	1.9 (0.9)	389.5	− 0.89*** [− 0.97; − 0.6]	− 0.96*** [− 0.99; − 0.72]	− 0.85*** [− 0.94; − 0.67]	0.1 [− 0.1; 0.29]	0.26* [0.05; 0.45]	0.27** [0.08; 0.44]
Resilience (CD-RISC-25)	71 (19)	59 (21.2)	65 (17.5)	76 (15)	46.7	0.42*** [0.27; 0.55]	0.22*** [0.11; 0.32]	− 0.18* [− 0.32; − 0.03]	− 0.23* [− 0.4; − 0.05]	− 0.57*** [− 0.72; − 0.38]	− 0.4*** [− 0.55; − 0.24]
Absorption (TAS)	2.4 (1)	3 (1)	2.8 (0.8)	3.5 (1)	91.2	− 0.39*** [− 0.52; − 0.23]	− 0.28*** [− 0.38; − 0.18]	− 0.63*** [− 0.73; − 0.5]	0.15 [− 0.04; 0.33]	− 0.34** [− 0.52; − 0.14]	− 0.48*** [− 0.62; − 0.3]
Self-esteem (RSE)	3.2 (0.8)	2.5 (0.9)	2.8 (0.7)	3 (0.9)	65.6	0.53*** [0.38; 0.66]	0.33*** [0.22; 0.43]	0.14 [− 0.02; 0.29]	− 0.28** [− 0.45; − 0.09]	− 0.43*** [− 0.6; − 0.23]	− 0.19* [− 0.35; − 0.01]
Self-concept clarity (SCCS)	4.5 (0.7)	3 (1.4)	3.7 (1.3)	3.3 (1.7)	157.9	0.73*** [0.62; 0.81]	0.52*** [0.42; 0.6]	0.55*** [0.4; 0.66]	− 0.32*** [− 0.47; − 0.14]	− 0.2 [− 0.39; 0.01]	0.1 [− 0.08; 0.28]

Medians (with IQRs) are shown. We performed Kruskal–Wallis tests (all *p* values < 0.001), which were followed up by pairwise Mann–Whitney tests. For the comparisons, Cliff’s Delta is shown as an estimate of effect size with 95% confidence intervals in brackets. Pairs in the header indicate which clusters are compared: low = low schizotypy; high = high schizotypy; disorg = low positive, high disorganised and intermediate interpersonal schizotypy; pos = high positive, intermediate disorganised and low interpersonal schizotypy.

***p < 0.001; **p < 0.01; *p < 0.05.

The four-cluster solution was better suited to address our research question as it yielded a small group characterised by remarkably high levels of positive schizotypy but only intermediate levels of disorganisation and a rather low extent of negative schizotypy. Additionally, the four-cluster solution was superior in terms of internal validity (see Supplementary Fig. S3), although silhouette width was relatively low, which suggests that the clusters were not clearly separated.

Ratio of males ranged from 26 to 34% and clusters did not differ significantly in terms of sex ratio (χ^2^(3) = 3.8, *p* = 0.284). However, clusters differed with respect to age (χ^2^(3) = 13.74, *p* = 0.003). Post-hoc Mann–Whitney tests indicated that age was significantly higher in the low schizotypy cluster (median = 23, IQR = 10) as compared to the disorganised cluster (median age = 21, IQR = 4) (p = 0.001, Cliff’s Δ = 0.19), and the positive schizotypy cluster (median = 21.5, IQR = 5.75) (*p* = 0.043, Cliff’s Δ = 0.16). No other differences were significant (all *p* values > 0.16). Median age in the high schizotypy cluster was 22 years (IQR = 3). All subsequent analyses were repeated adjusting for age and sex and these results are reported in the supplementary materials (see Supplementary Table S1, Supplementary Fig. S4).

### Comparing the schizotypy clusters in terms of resilience, absorption, and valuation and integrity of the self

**Figure 2:**
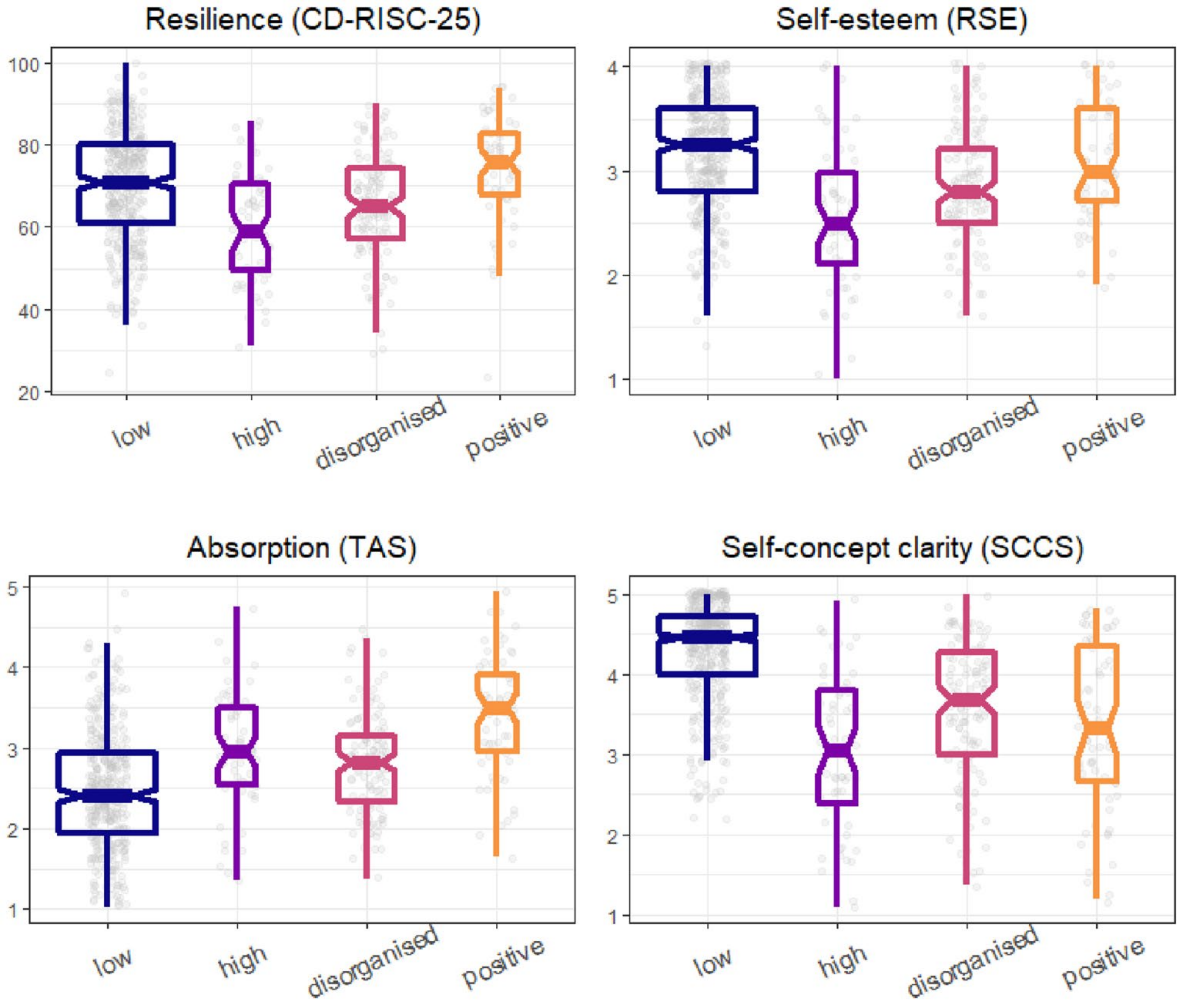
Comparison of the schizotypy clusters. See Table 2 for the results of the statistical analyses. Note that the points are jittered to facilitate visibility.

Then, we compared the clusters in terms of resilience, absorption, self-esteem, and self-concept clarity. Critically, the two competing versions of the adaptive schizotypy hypothesis give different predictions about the positive schizotypy group: according to the strong version, this group should have no impairment, while the moderate version expects a mix of impairment and traits promoting well-being. The differences are visualised in Fig. 2. Descriptive statistics and the results of the statistical analyses are shown in Table 2. The positive schizotypy cluster had the highest and the high schizotypy cluster had the lowest resilience scores. Crucially, the positive schizotypy cluster was more resilient even relative to the low schizotypy cluster. Each schizotypy cluster had significantly higher absorption scores than the low schizotypy cluster. Importantly, the positive schizotypy cluster was again superior to every other cluster. The mixed schizotypy cluster had significantly worse self-esteem, relative to every other cluster. The positive schizotypy cluster had better self-esteem than the mixed and the disorganised-interpersonal cluster, while it did not differ significantly from the low schizotypy cluster. Self-concept clarity strongly differentiated the low schizotypy cluster from the other clusters; in addition, the mixed schizotypy cluster demonstrated significantly lower self-concept clarity than the disorganised-interpersonal group. Adjusting for age and sex did not essentially change the pattern of findings (see Supplementary materials). To sum up, the positive schizotypy cluster had remarkably high resilience and absorption, while it did not show a significant decrement in self-esteem, relative to the low schizotypy cluster. At the same time, it showed impaired self-concept clarity.

## Discussion

The aim of our study was to compare a strong and a moderate version of the adaptive schizotypy hypothesis^42^ by examining a set of indicators related to well-being and impairment in a group characterised by high positive and low negative schizotypy. We collected data in a large sample of university students using a set of well-established, reliable, and valid instruments. We extracted four clusters: a larger, overall low and a smaller, overall high schizotypy cluster, and two smaller clusters with intermediate levels of schizotypy. One of the latter groups was characterised by high disorganised schizotypy, intermediate negative schizotypy and lower positive schizotypy, while the other group, termed the positive schizotypy cluster, showed an inverse pattern: in this group we observed high positive schizotypy, intermediate disorganisation and low negative schizotypy. Comparison of the clusters supported the moderate version of the adaptive schizotypy hypothesis: the positive schizotypy cluster showed intact self-esteem, very high levels of absorption that may promote creativity^49^ and spirituality^51^ and its high resilience can preserve mental health in the face of adversity^65,77^. Yet, this group also demonstrated a sign of dysfunctional personality organisation: in the positive schizotypy group, self-concept clarity was drastically impaired.

Our findings are in line with the literature showing an association of positive schizotypy with indicators of resilience. Positive schizotypy predicts creativity in the arts^33,47–49^, and it correlates with increased capacity to experience and express pleasure^36^ and with enhanced effort to obtain rewards^85^. Further underscoring the protective potential of positive schizotypy, it predicts personal and family history of better mental health, over and above the negative effect of disorganised schizotypy^86^.

One may speculate that the contrasting profiles of disorganised-interpersonal and the positive schizotypy clusters indicate differential vulnerability for motivational deficit vs. cognitive distortions/positive symptoms in schizophrenia spectrum disorders, respectively. Relatedly, groups that resemble our clusters have been found among patients. A study has reported a “Kraepelinian Schizophrenia” latent class with elevated disorganisation, negative, and positive symptoms, mania and depression (paralleled by our overall high schizotypy cluster), an “Affective Psychosis” latent class with average disorganisation and average negative symptoms, but high levels of mania, depression and positive symptoms (analogous to the high positive cluster in our study), and a “Deficit Nonpsychosis” class with higher disorganisation and negative symptoms, but below-average mania, depression and positive symptoms (resembled by the disorganised-interpersonal cluster we found)^87^. Whether the clusters identified here are predictive of risk for developing the above-mentioned symptom-profiles is to be examined by longitudinal studies. Nevertheless, available follow-up data that used continuous scores to predict risk implicates that the clusters indeed have predictive validity. In the Chapman’s 10-year longitudinal study, positive schizotypy uniquely and specifically predicted psychotic, depressive and manic disorders, while negative schizotypy had a unique relationship with schizoid personality, whereas both traits predicted being diagnosed with any schizophrenia-spectrum disorder^14^.

The study has several limitations. First, recruiting students could have introduced a sampling bias, as attending university demands a certain level of global and academic functioning, intelligence, and socio-economic status. Individuals developing schizophrenia-spectrum disorders have been shown to have a particularly high rate of drop-out from high school^88^. Therefore, it may seem likely that those who are more impaired and are at higher risk for developing such disorders are underrepresented among university students. On the other hand, we detected a high schizotypy cluster in our sample that had high levels of all dimensions of schizotypy and showed the greatest impairment in terms of self-esteem and resilience. Relatedly, longitudinal studies suggest that a combination of high positive and high negative schizotypy predicts the greatest impairment and the highest risk of psychotic disorders at a 10-year follow-up^13,14^. Nevertheless, positive schizotypy predicts unique aspects of impairment such as drug or alcohol abuse, mood disorders and suicide attempts^14^, leaving the possibility open that individuals with extremely high levels of positive schizotypy—comparable to the positive schizotypy cluster—and low resilience are too impaired to attend university. Future studies should attempt to replicate the findings in demographically more heterogeneous community samples. Second, we relied exclusively on self-report measures, which could be prone to recall and social desirability biases. Finally, the items of the SPQ-BR are based on the diagnostic criteria for schizotypal personality disorder, thus, they are more inclined to be worded in a way that captures impairing aspects of schizotypy (e.g. ‘I often feel that others have it in for me’), which could confound positive schizotypy with high distress and preclude detection of a high positive schizotypy group with no signs of maladaptation or impairment. This might be remedied by using somewhat less clinically worded indicators of positive schizotypy, such as the Oxford-Liverpool Inventory of Feelings and Experiences^89^, or, an instrument which separately assesses the intensity and the distressing nature of positive schizotypal experiences, such as the Peters et al. Delusions Inventory^90^ or the Cardiff Anomalous Perceptions Scale^91^.

Our study provides novel insight about whether positive schizotypy can have purely beneficial, non-pathological forms. Its strengths include the use of a large undergraduate sample and a set of well-established and reliable questionnaires. Moreover, the extraction of four clusters was supported by data-driven indicators of cluster goodness, not just by theoretical considerations. Critically for our research question, we have detected a group of individuals with high positive, low negative, and intermediate disorganised schizotypy. This group had a profile that strongly contrasted with groups that showed either high or low levels of all dimensions of schizotypy. It was characterised by extremely high levels of traits related to increased well-being such as resilience and absorption, but also had impaired self-concept clarity, which is known to signal a proneness to mental health problems. Our findings speak for a moderate version of the adaptive schizotypy hypothesis, which recognises both the benefits and vulnerabilities associated with positive schizotypy.

## Supplementary Information

Supplementary Information.
