# Supplementary material for: Adaptive and maladaptive features of schizotypy clusters in a community sample
Source: Sci Rep. 2021 Aug 17;11:16653. doi: 10.1038/s41598-021-95945-0 (PMC8371157; doi:10.1038/s41598-021-95945-0)
Supplement: Supplementary file 1 — Supplementary Information. [file 41598_2021_95945_MOESM1_ESM.doc]

Adaptive and maladaptive features of schizotypy clusters in a community sample

Bertalan Polner^a*^, Ernő Hupuczi^b^, Szabolcs Kéri^a,c,d^, János Kállai^b^

^a^Department of Cognitive Science, Budapest University of Technology and Economics, Budapest, Hungary

^b^Cognitive Neuroscience Research Group, Institute of Behavioral Sciences, Medical Faculty, University of Pécs, Pécs, Hungary

^c^National Institute of Psychiatry and Addictions, Budapest, Hungary

^d^Department of Physiology, University of Szeged, Szeged, Hungary

Corresponding author: Bertalan Polner, [bpolner@edu.bme.hu](mailto:bpolner@edu.bme.hu)

# Supplementary materials

## Clustering tendency of the dataset


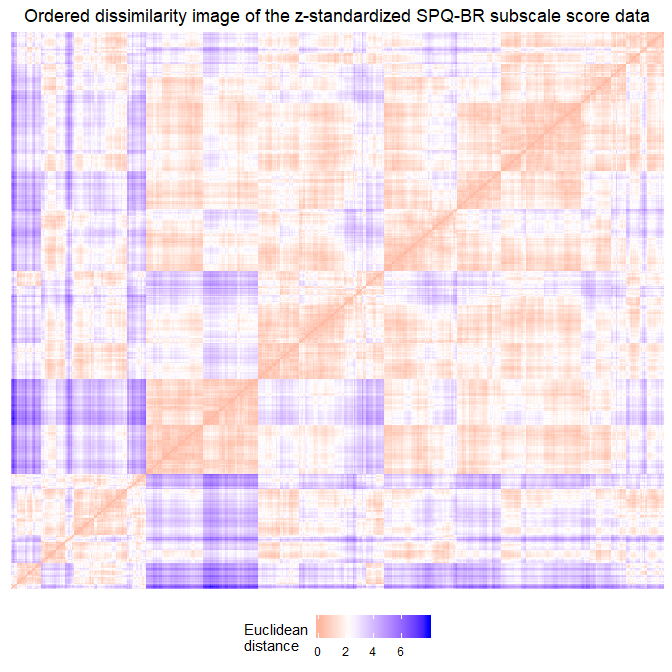


**Supplementary Figure 1.** Ordered dissimilarity image of the data.

## Choosing linkage measure for hierarchical clustering


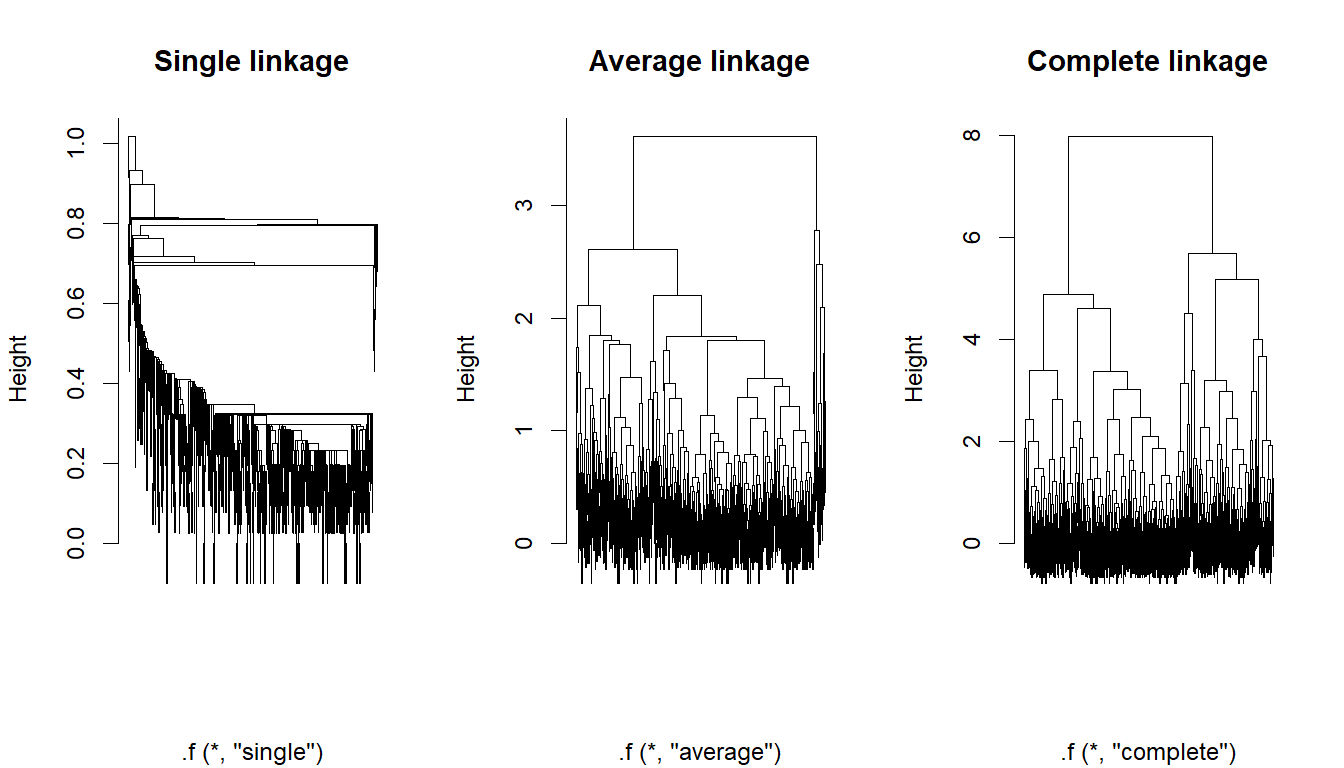


**Supplementary Figure 2.** Dendrograms for hierarchical clustering on the z-standardized cognitive-perceptual, interpersonal and disorganised SPQ-BR subscale scores, using Euclidean distance.

## Internal validation of the clusters

In order to assess the internal validity of the clusters, we computed connectivity, the Dunn-index, silhouette width (Brock et al., 2011) and the S_Dbw index (Liu et al., 2010) for solutions including from 2 up to 6 clusters. *Connectivity* indicates the extent to which near data points get assigned to different clusters and should be minimised. We computed connectivity using 10 neighbours. The *Dunn-index* is the ratio of the minimum distance between data points in different clusters to the maximum distance between data points assigned to the same cluster and should be maximised. *Silhouette width* is the average of silhouette values in the whole dataset. Intuitively speaking, the silhouette value indicates whether a data point is closer to data points in its cluster vs. data points in the next nearest cluster. The silhouette value ranges between -1 and 1: values near 1 indicate excelling clustering of a data point, values near -1 suggest “wrong” clustering, while values around 0 suggest uncertain clustering. Thus, silhouette width should be maximised. Finally, we computed the *S_Dbw index*, as it has been suggested to be a well-performing internal validity measure of clustering in various scenarios (Liu et al., 2010). The S_Dbw index combines between-cluster density and within-cluster compactness, and should be minimised.


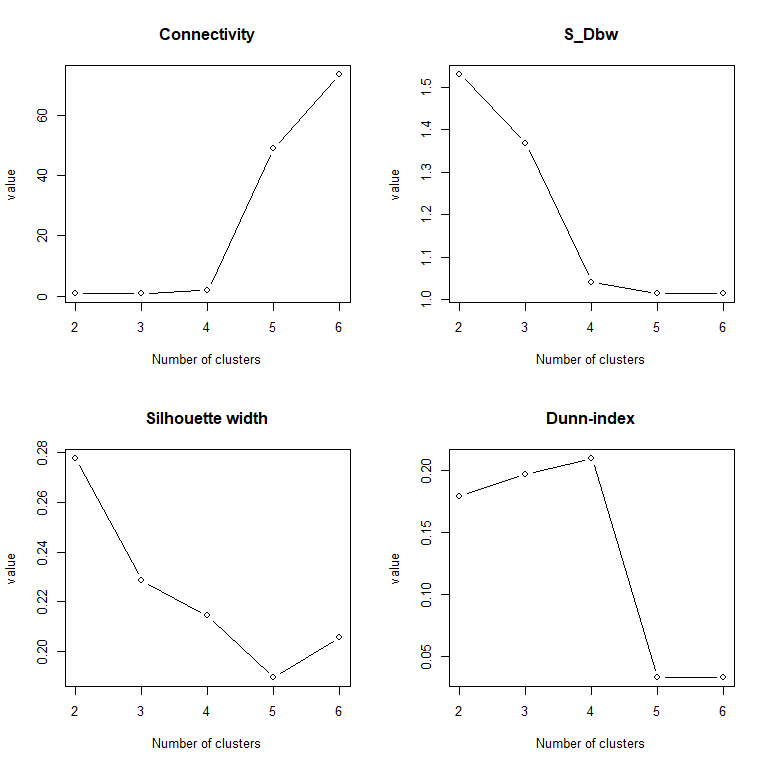
 **Supplementary Figure 3.** Internal validity of various number of clusters obtained with hierarchical clustering, using Euclidean distance and complete linkage. Connectivity and S_Dbw should be minimised, and silhouette width and the Dunn index should be maximised.

## Comparison of the clusters adjusted for age and sex

| Dependent variable | low | high | disorg | pos | χ2(3) | low vs. high | low vs. disorg | low vs. pos | high vs. disorg | high vs. pos | disorg vs. pos |
| --- | --- | --- | --- | --- | --- | --- | --- | --- | --- | --- | --- |
| **Interpersonal schizotypy  (SPQ-BR)** | -0.3 (1.1) | 1.5 (0.5) | 0.3 (0.7) | -0.4 (0.5) | 197.0 | **-0.96*** [-0.99; -0.9]** | **-0.42*** [-0.53; -0.36]** | 0.06 [-0.1; 0.16] | **0.94*** [0.9; 0.97]** | **0.99*** [0.97; 1]** | **0.58*** [0.43; 0.72]** |
| **Cognitive-perceptual schizotypy (SPQ-BR)** | -0.3 (0.6) | 0.4 (0.5) | -0.1 (0.5) | 0.8 (0.7) | 212.9 | **-0.77*** [-0.85; -0.68]** | **-0.25*** [-0.37; -0.17]** | **-0.89*** [-0.95; -0.74]** | **0.71*** [0.6; 0.81]** | **-0.36*** [-0.53; -0.15]** | **-0.86*** [-0.92; -0.79]** |
| **Disorganised schizotypy  (SPQ-BR)** | -0.5 (0.6) | 0.9  (1) | 0.8 (0.7) | 0.4 (0.8) | 371.5 | **-0.87*** [-0.97; -0.6]** | **-0.94*** [-0.99; -0.72]** | **-0.83*** [-0.94; -0.67]** | 0.1 [-0.1; 0.29] | **0.25* [0.05; 0.45]** | **0.26** [0.08; 0.44]** |
| **Resilience  (CD-RISC-25)** | 2.3 (18.7) | -8.6 (19.9) | -3.1 (16) | 7.5 (15.1) | 43.6 | **0.41*** [0.27; 0.55]** | **0.19*** [0.11; 0.32]** | **-0.19* [-0.32; -0.03]** | **-0.23* [-0.4; -0.05]** | **-0.58*** [-0.72; -0.38]** | **-0.39*** [-0.55; -0.24]** |
| **Absorption  (TAS)** | -0.3  (1) | 0.3  (1) | 0.2 (0.8) | 0.8  (1) | 90.3 | **-0.38*** [-0.52; -0.23]** | **-0.29*** [-0.38; -0.18]** | **-0.63*** [-0.73; -0.5]** | 0.16 [-0.04; 0.33] | **-0.34** [-0.52; -0.14]** | **-0.48*** [-0.62; -0.3]** |
| **Self-esteem  (RSE)** | 0.2  (0.8) | -0.5 (0.8) | -0.2 (0.7) | -0.1 (1) | 59.0 | **0.52*** [0.38; 0.66]** | **0.31*** [0.22; 0.43]** | 0.11 [-0.02; 0.29] | **-0.28** [-0.45; -0.09]** | **-0.43*** [-0.6; -0.23]** | **-0.18* [-0.35; -0.01]** |
| **Self-concept clarity  (SCCS)** | 0.4  (0.8) | -0.8 (1.1) | -0.3 (1.2) | -0.4 (1.5) | 136.6 | **0.71*** [0.62; 0.81]** | **0.46*** [0.42; 0.6]** | **0.51*** [0.4; 0.66]** | **-0.32*** [-0.47; -0.14]** | -0.21 [-0.39; 0.01] | 0.1 [-0.08; 0.28] |

**Supplementary Table 1.** Statistical comparison of the clusters adjusted for age and sex. Medians (with IQRs) are shown. After adjusting the dependent variables for age and sex effects with linear regressions, we performed Kruskal-Wallis tests (test statistic reported in the second column, all *p* values < 0.001), which were followed up by pairwise Mann-Whitney tests. For the comparisons, Cliff’s Delta is shown as an estimate of effect size with 95% confidence intervals in brackets. Pairs in the header indicate which clusters are compared: low = low schizotypy; high = high schizotypy; disorg = low positive, high disorganised and intermediate interpersonal schizotypy; pos = high positive, intermediate disorganised and low interpersonal schizotypy. *** *p* < 0.001; ** *p* < 0.01; * *p* < 0.05


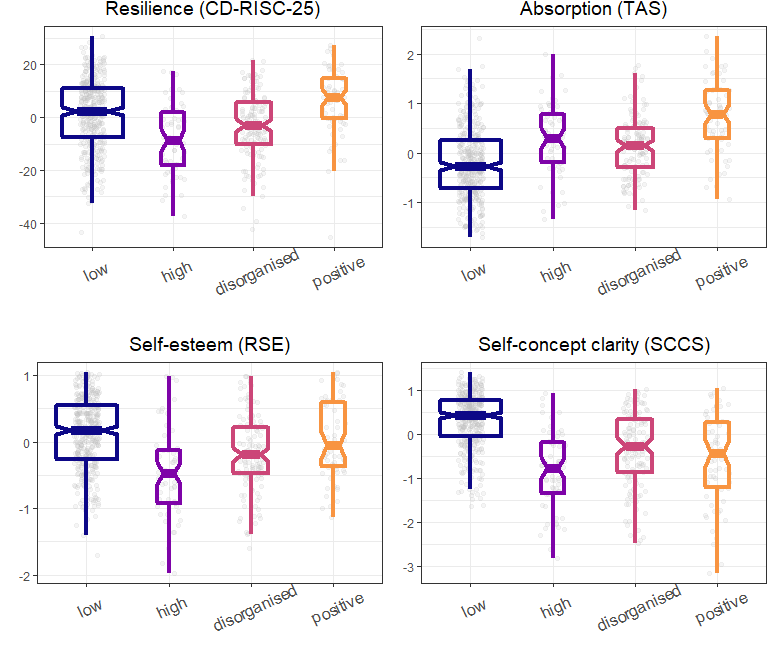


**Supplementary Figure 4.** Comparison of the schizotypy clusters, with the dependent variables adjusted for age and sex. See Supplementary Table 1. for the results of the statistical analyses. Note that the points are jittered to facilitate visibility.
